# Dead Crow Density and West Nile Virus Monitoring, New York

**DOI:** 10.3201/eid1109.040712

**Published:** 2005-09

**Authors:** Millicent Eidson, Kate Schmit, Yoichiro Hagiwara, Madhu Anand, P. Bryon Backenson, Ivan Gotham, Laura Kramer

**Affiliations:** * New York State Department of Health, Albany, New York, USA

**Keywords:** West Nile virus, epidemiology, forecasting, flaviviruses, research

## Abstract

New York State used the health commerce system to monitor the number of West Nile virus (WNV) human disease cases and the density of dead crows. In each year from 2001 to 2003 and for the 3 years combined, persons living in New York counties (excluding New York City) with elevated weekly dead crow densities (above a threshold value of 0.1 dead crows per square mile) had higher risk (2.0–8.6 times) for disease caused by WNV within the next 2 weeks than residents of counties reporting fewer dead crows per square mile. This type of index can offer a real-time, relatively inexpensive window into viral activity in time for prevention and control. Changes in reporting, bird populations, and immunity may require that thresholds other than 0.1 be used in later years or in other areas.

## Abstract 2

Persons in counties with high dead crow densities had elevated risk for disease.

Controlling West Nile virus (WNV) and other vectorborne pathogens requires identifying areas of risk as early as possible. Ideally, risk indices should be relatively inexpensive and easy to implement, should provide timely and accurate forecasts of risk, and should not trigger expensive or controversial control measures when the actual risk is low. WNV infections in >200 species of birds (1) and associated bird deaths have been reported from multiple locations in the Western Hemisphere (2–8). Studies indicate that dead crow (*Corvus* spp.) reports have a number of advantages, before or without laboratory confirmation (9–16): 1) crows appear to be extremely sensitive to WNV infection and have a high case-fatality rate (6,17), which makes WNV transmission to crows relatively easy to detect; 2) crows are widely distributed, large, and generally easy to recognize; 3) crows have high mean viremia levels (17) and a high reservoir competence index (1), which indicates that they are a good source of virus for mosquito infections and local disease amplification; and 4) no resources or time are required for bird or sample collection, processing, and testing.

Analyses of data from the northeastern United States in 2000 and 2001 found that counties with high dead crow densities (DCD, dead crows per square mile) early in the season were significantly more likely to have a case of WNV disease in a human (18). In New York in 2000, the number of human disease cases by county was more strongly associated (r = 0.92) with DCD than with the number of WNV-positive birds or with the number of sightings of all bird species (19). In addition, weekly DCD increased several weeks before the onset of cases in humans (19,20). In New York counties with no human cases, DCD never exceeded 0.1. In counties with 1 or 2 cases of human disease, DCD exceeded 0.1 before human case onset and reached 1.4. In Richmond County (Staten Island), with 10 human cases, DCD exceeded 1.5 before disease onset in humans and peaked at 7. This article evaluates New York's real-time use since 2000 of the weekly, county-level DCD index as an indicator of human WNV disease risk, with a signal level of 0.1.

## Methods

The New York State Department of Health (NYSDOH) developed a Web-based secure health commerce system that supports all of its information interchange with public health and healthcare communities (21). In the spring of 2000, NYSDOH implemented a statewide, integrated WNV surveillance system on health commerce in response to the 1999 emergence of WNV. The system includes real-time surveillance components for humans, mammals, birds, and vectors and allows local health departments, the state dead bird hotline, and laboratories to enter and retrieve surveillance data in real time for disease tracking (22). Although sightings of dead birds of any species are reported by private citizens and agencies, dead crow sightings are emphasized in automated summary tables and charts based on their utility in previous studies. Because New York City developed its own WNV surveillance system to monitor dead bird reports, New York City data have not been included in this follow-up study. By using real-time surveillance data, system users can press a button to immediately generate a DCD calculation and graph for each county in the state for a specific period of interest. For this study, the weekly DCD was graphed for each county during a season to monitor trends over time, and human cases were added when they met the 2001 Centers for Disease Control and Prevention (CDC) case definitions for a confirmed or probable case (23).

**Figure 1:**
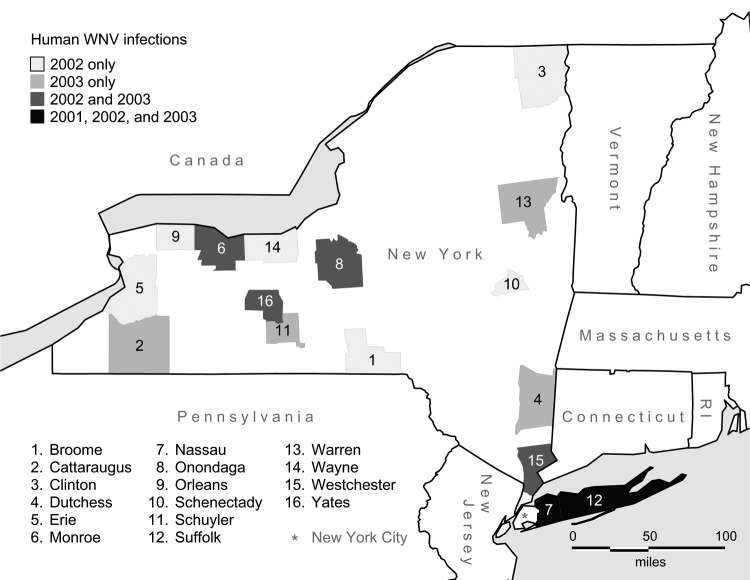
New York counties with laboratory-confirmed cases of human West Nile virus disease, 2001–2003.

According to CDC case definitions, New York (excluding New York City) had 6 (1 excluded from study) confirmed or probable human cases of WNV disease in 2001, 53 (3 excluded) in 2002, and 40 (3 excluded) in 2003. Reasons for case exclusion were occupational exposure (2001), infection by blood-transfusion (2002), date of onset after dead bird surveillance had abated (2002, 2003), intrauterine transmission (2002), and travel outside of New York at likely time of infection (2 cases in 2003). The 92 human cases included in the study were widely distributed in New York except for the sparsely populated north-central region (Figure 1).

The Cochran-Mantel-Haenszel (CMH) option of the "freq" procedure in SAS (SAS System for Windows V8, SAS Institute, Cary, NC, USA) was used to calculate point and interval estimates of WNV disease risk, depending on DCD >0.1 in a person's county of residence. Data from each week with onset of a human case were included in the analysis (8/19–9/22 in 2001, 7/28–10/5 in 2002, and 8/3–9/27 in 2003). For each week, a table was constructed comparing the week's human cases per population in counties with a "signal" of DCD >0.1 and the human cases per population in counties with no signal. For example, 9 patients had disease onset in week 35 of 2003; 8 of the patients resided in counties with DCD >0.1 in the previous 2 weeks and 1 of whom did not (Table 1). (Note that in other weeks the total population in the 2 categories is different, depending on which counties had high DCD in the previous 2 weeks.)

**Table 1:** Example of 2×2 table constructed for chi-square analyses in this study*

Week 35, 2003	Counties with DCD >0.1 in past 2 wks	Counties with DCD <0.1 in past 2 wks
No. cases with disease onset during week	8	1
Total population	4,422,461	6,545,718

*Data for each week with human West Nile virus disease cases in 2001–2003 were pooled to calculate risk for disease occurring within 2 weeks of elevated dead crow density (DCD) in county relative to risk in counties without elevated DCD in the past 2 weeks.

The CMH chi-square statistic was used to compare the incidence (risk) of WNV disease in the DCD signal areas with the incidence in the non-DCD signal areas over all the weeks of this study. As implemented by SAS (24), this procedure pools data across strata (in this case, across weeks), determines a p value for the difference in incidence between the 2 exposure categories, and estimates a single relative risk for the exposed versus the unexposed population across all weeks (including those weeks when fewer cases resulted in risk estimates that would not be considered meaningful).

In New York's dead crow surveillance system, an increase in a county's crow death reports assumes an increase in the number of infected mosquitoes able to transmit virus to both crows and humans. Since the time between WNV exposure (mosquito bite) and human disease onset is 2–14 days (25), we assumed that <2 weeks could pass between the exposure of interest (high DCD in the county of residence) and disease onset. For this reason, CMH-pooled risk estimates were calculated separately for 3 exposure periods, defined as the county having a density signal 2 weeks before the onset week of the human case, 1 or 2 weeks before the onset week of the human case, or in the onset week of the human case or 1–2 weeks before.

## Results

**Figure 2:**
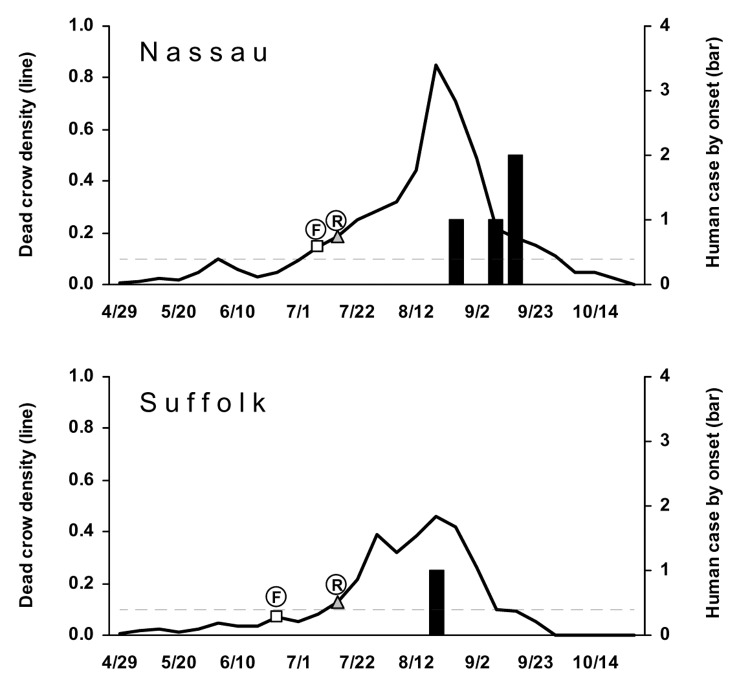
Dead crow densities (DCD, dead crows per square mile) and number of cases of human West Nile virus (WNV) disease, by week, 2001. Horizontal dashed line indicates DCD = 0.1. F, date that the first bird with confirmed WNV infection was found; R, date that the laboratory result of the first bird with WNV infection was reported.

For the 2 Long Island counties with human cases of WNV disease in 2001, weekly DCD >0.1 were seen more than 1 month before onset of human cases (Figure 2). DCD increased before the first WNV-positive bird was reported (1–3 weeks after it was found). However, the highest peaks in weekly DCD occurred after viral activity was confirmed and may have been influenced by increased interest in reporting dead crows after the media had reported WNV in the area. For other New York counties, weekly DCD remained lower (<0.1), and no human cases were detected.

**Figure 3:**
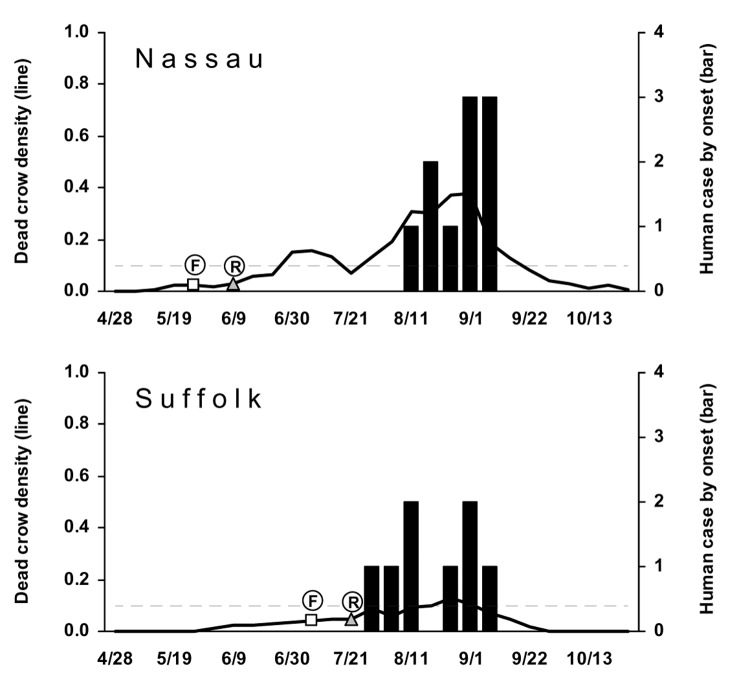
Dead crow densities (DCD, dead crows per square mile) and number of cases of human West Nile virus (WNV) disease, by week, 2002. Horizontal dashed line indicates DCD = 0.1. F, date that the first bird with confirmed WNV infection was found; R, date that the laboratory result of the first bird with WNV infection was reported.

In 2002, Long Island (Nassau and Suffolk counties) had the most human cases (Figure 3). However, almost as many human cases were reported for Erie and Broome counties further north. Eight other counties reported 1–4 cases. In general, DCD >0.1 occurred several weeks before the onset week of the first human case in most counties, with the peak density around the period of the human case onset, except in sparsely populated rural counties (Clinton, Orleans, Wayne, and Yates), which reported 1 human case each. Suffolk County is notable for its relatively lower weekly DCD. Two counties had DCD >0.1 without human cases. The first WNV-positive bird of the season was typically reported 1–4 weeks after the bird was found. Sharp increases in DCD immediately after the report of the first WNV-positive bird were not generally noted in 2002.

**Figure 4:**
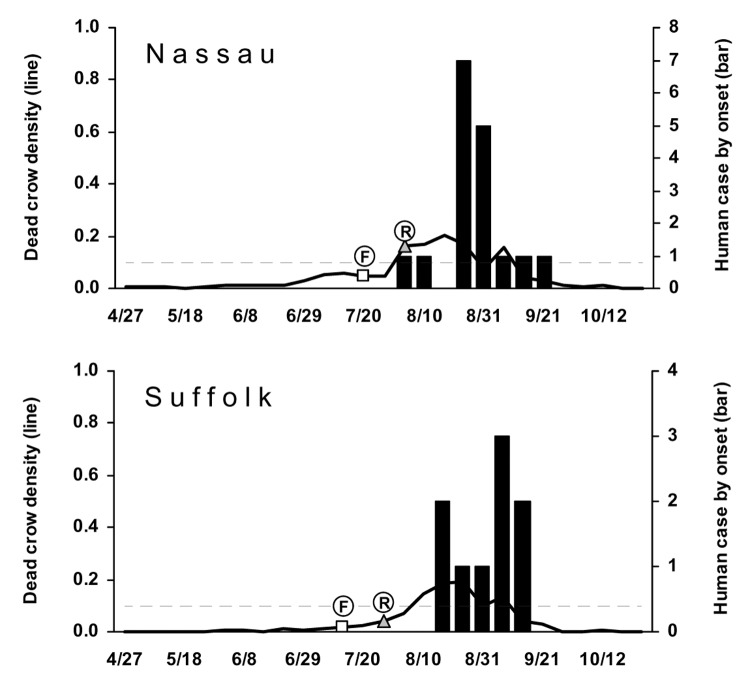
Dead crow densities (DCD, dead crows per square mile) and number of cases of human West Nile virus (WNV) disease, by week, 2003. Horizontal dashed line indicates DCD = 0.1. F, date that the first bird with confirmed WNV infection was found; R, date that the laboratory result of the first bird with WNV infection was reported.

A similar pattern was seen in 2003, with Long Island (Nassau and Suffolk counties) again leading the number of human cases (Figure 4). All 3 counties with >1 human case had DCD >0.1 in the week of, or the weeks before, the first human case onset. A similar pattern was seen for Monroe County with 1 human case. Onondaga County, with 1 human case, had a weekly DCD >0.1 after the human case onset. The DCD approached 0.1 in Dutchess County 2 weeks before the week of the human case onset. The DCD remained low in the sparsely populated counties (Cattaraugus, Schuyler, Warren, and Yates) with 1 human case. Two counties had DCD >0.1 without human cases. In 2003, laboratory confirmation of viral activity in a dead bird was available 1–2 weeks after the bird was found. DCD increases after those reports were observed for some, but not all, counties.

For each year and for the 3 years combined, the CMH pooled estimate of risk for WNV disease among residents of counties with DCD >0.1 was >2 times the risk among residents of counties with DCD <0.1 (Table 2). Relative risks were highest in 2001; residents of counties with elevated DCD had 7.6–8.6 times the risk of contracting WNV disease than residents of counties with lower DCD. Relative risks were lower in 2002 (2.0–2.3) but increased in 2003 (5.3–6.5). During the 3-year period, residents of counties with elevated DCD had 3.4–3.8 times the risk of contracting WNV disease within the next 2 weeks than residents of counties reporting fewer dead crows per square mile.

**Table 2:** Risk for West Nile virus disease in counties with elevated dead crow density (DCD) in the 2 weeks before disease onset*

Counties with elevated DCD†	2001 RR (95% CI)	2002 RR (95% CI)	2003 RR (95% CI)	2001–2003 RR (95% CI)
2 weeks before onset	8.6 (1.8–41.8)	2.2 (1.1–4.6)	5.4 (2.1–14)	3.5 (2.0–6.0)
1 or 2 weeks before onset	7.9 (2.9–19.1)	2.3 (1.1–4.8)	6.5 (2.6–16.3)	3.8 (2.2–6.6)
0, 1, or 2 weeks before onset	7.6 (1.6–36.8)	2.0 (0.95–4.4)	5.3 (2.2–12.8)	3.4 (1.9–5.9)

*Versus residents of counties without elevated DCD in the previous 2 weeks; risk is calculated for exposure to elevated DCD only 2 weeks before, 1 or 2 weeks before, and during the same week or 1 or 2 weeks before week of onset. SAS provides risk estimates by 2 methods; because zero values occur in 2×2 tables for some weeks, we report results by the adjusted logit method, which uses a 0.5 correction for zero-value cells. RR, relative risk; CI, confidence interval. †Weekly DCD >0.1.

## Discussion

As in 2000 (19,20), increases in weekly number of dead crows per square mile were found in most New York counties several weeks before onset of human cases in 2001 through 2003. Persons in counties with DCD >0.1 were notably more likely (2–9 times) to contract WNV disease in the next several weeks. Applying this signal statewide (including New York City) over all 4 years (2000–2003), we found that 148 (91%) of 163 human patients resided in counties with this signal during or before the week of illness onset. This result supports findings of increased DCD in association with human WNV cases in other areas (12,15,18). The specific level of the signal (>0.1 dead crows per square mile per week) may not be applicable in all counties or in New York in the future, if reporting changes, crow populations are reduced, or crows become more resistant to infection.

For counties with >1 patient, the density index rarely failed to forecast the increased risk. Suffolk County in 2002 was a notable exception. If continuous WNV activity led to immunity in crows, this type of signal would be less effective. However, to date the case-fatality rate is still believed to be high, since few crows are found alive with antibody to indicate immunity (6,17). Alternatively, if large-scale death from WNV decreases the crow population, fewer crow deaths would be seen and reported. Data indicate a large die-off of crows in Suffolk County: 5,788 in 2000 and 2,953 in 2001. In 2002, dead crow reports dropped to 883 and were at 939 for 2003.

Weekly county-level DCD was less useful at forecasting the occasional single human case in less-populated counties. Rural areas have fewer persons to report dead crows and fewer persons to become infected with WNV, even when infected mosquitoes and birds are in the area. In any area, including those with few crows, monitoring all dead bird sightings would provide more reports to use for possible WNV tracking, and these sightings should be studied in areas with few crow reports. The value of having more reports may be offset by the lower case-fatality rate in other species. However, even in Florida, where dead birds from the dove family (order Columbiformes) were reported more frequently than dead corvids, the number of dead crow reports, adjusted for human population, was higher in focal areas of WNV transmission, and crow deaths peaked at the same time or before some (but not all) human cases (13).

On rare occasions, the weekly DCD signal provided a false indication of increased risk in a county without human cases. NYSDOH emphasizes reporting and preventing neurologic cases; milder cases of disease such as West Nile fever could have occurred undetected in those counties. A previous study found a pattern of decreasing DCD after mosquito control (19), which indicates that control activities could have reduced the risk, or that differences in mosquito species and their host preferences may have existed. In developing risk indicators that can be relied upon for determining prevention and control actions, occasional false signals may not be a problem if the consequence is increased surveillance and education. If signals trigger resource-intensive control programs, however, false-positive signals may be more problematic.

An important issue is how to maintain the public's interest in reporting if bird testing is reduced or stopped after the virus is confirmed in an area. If dead crows are unreported, because people are either not in the area to find them or not interested in reporting them, DCD will be less useful for monitoring risk. A disadvantage of the county-level approach presented in these analyses is that it does not allow identification within a county of the higher-risk areas. Geographic information system approaches to identify geographic clustering of dead crow or dead bird reports, with or without laboratory confirmation, can help locate areas of risk (14,15,26,27). Such studies are underway in New York (28,29), but they are more resource intensive, and thus more difficult to institute in real time, than the county-level density index.

With the number of human WNV cases increasing to nearly 10,000 in the United States in 2003 (30), the need for surveillance tools to determine when to use costly prevention and control measures has increased. The rapid spread of emerging diseases, such as WNV disease, and the potential use of disease agents for bioterrorism have shown the need to develop real-time surveillance tools, especially for detecting disease activity before laboratory-confirmed cases are reported.

Examples of real-time tools include syndromic surveillance systems (31), most of which rely on indicators other than laboratory-confirmed cases. Although surveillance systems that use laboratory-confirmed cases will continue to have a critical role, a more rapid index of viral activity, such as New York's DCD index, may be useful in some situations. Even if increases in DCD provide an early warning of increasing viral activity and risk to humans, more research is required for systems that function well to share this information in real time, securely and confidentially, with appropriate public health partners who can adjust surveillance and control procedures (32–34).
